# ”We own the illness”: a qualitative study of networks in two communities with mixed ethnicity in Northern Norway

**DOI:** 10.1080/22423982.2018.1438572

**Published:** 2018-02-21

**Authors:** Anette Langås-Larsen, Anita Salamonsen, Agnete Egilsdatter Kristoffersen, Torunn Hamran, Bjørg Evjen, Trine Stub

**Affiliations:** ^a^ The National Research Center in Complementary and Alternative Medicine (NAFKAM), Department of Community Medicine, The Faculty of Health Sciences, UiT the Arctic University of Norway, Tromsø, Norway; ^b^ Regional Centre for Child and Youth Mental Health and Child Welfare (RKBU North), The Faculty of Health Sciences, UiT the Arctic University of Norway, Tromsø, Norway; ^c^ Department of Health and Care Sciences. Centre for Care Research, The Faculty of Health Sciences, UiT the Arctic University of Norway, Tromsø, Norway; ^d^ Centre for Sami Studies, The Faculty of Humanities, Social Sciences and Education, UiT the Arctic University of Norway, Tromsø, Norway

**Keywords:** Sami, ethnic mixed culture, folk medicine, traditional healing, traditional network, Siida

## Abstract

**Background**: When people in Northern Norway get ill, they often use traditional medicine. The global aim of this study was to examine the extended family networks’ function and responsibility in cases of illness in the family, in two Northern Norwegian communities with a population of mixed ethnicity.

**Methods**: Semi-structured individual interviews with 13 participants and 4 focus group interviews with total 11 participants were conducted. The text data was transcribed verbatim and analysed based on the criteria for content analysis.

**Results**: The participants grew up in areas where it was common to seek help from traditional healers. They were organized in networks and shared responsibility for the patient and they provided practical help and support for the family. According to the networks, health-care personnel should make room for the entire network to visit the patient in severe and life-threatening situations.

**Conclusion**: Traditional networks are an extra resource for people in these communities. The networks seem to be essential in handling and disseminating hope and manageability on an individual as well as a collective level. Health personnel working in communities with mixed ethnicity should have thorough knowledge of the mixed culture, including the importance of traditional network to the patients.

## Introduction

Social networks are often defined as long-lasting relationships between people They consist of two components, people and the relations between them [1]. A common social network in Norway is the *nuclear family* that includes the mother, father and children. This type of network represents at its best a source of safety and peace of mind, and also practical support [2].

**Figure 1.**
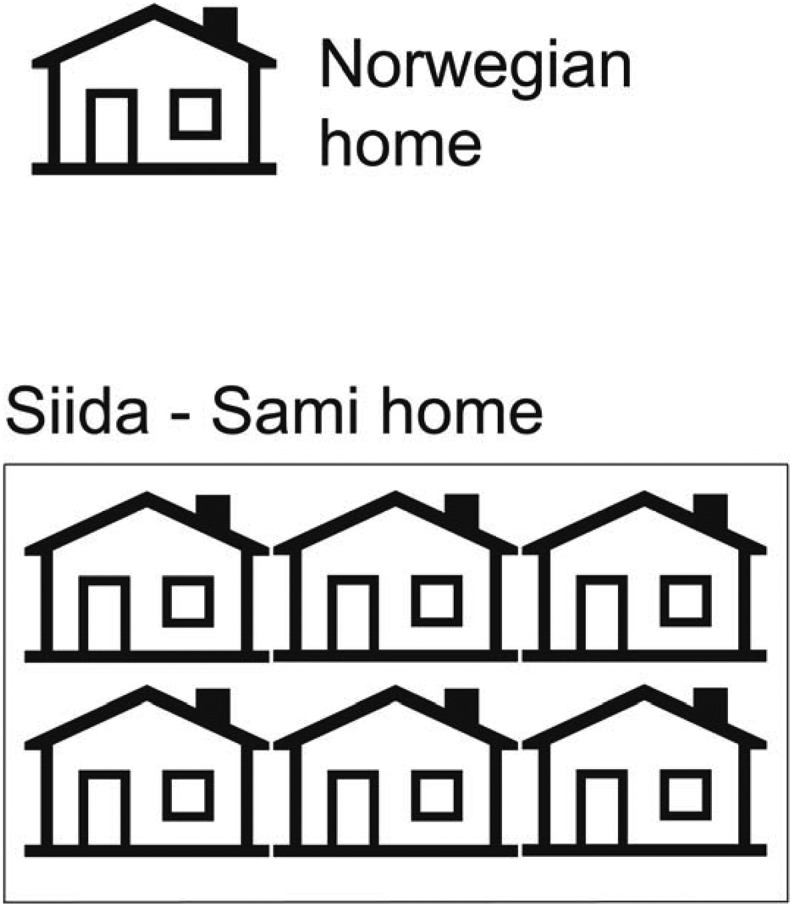
This figure illustrates the difference between the Norwegian nuclear family home and the Sami home that often includes an extended family network (*siida).*

According to Fugelli and Ingstad [2], who have examined the Norwegians´ experiences of good health, there is a positive correlation between help and support from family and friends and good health. Social networks often serve as buffers and may contribute to reducing strain caused by negative life events and stress [3]. Solid networks promote good physical as well as mental health, and research shows that one out of three would contact friends in difficult mental situations [4].

Many elderly people may have weaker social networks than younger people [3]. Elderly people who lack help and support from their families and friends have poorer quality of life, shorter lifetime and seek help from the public health service more often than elderly with strong family and neighbour networks [5]. Berkman and Syme [6] found that the mortality among people with weak social networks was twice as high compared to those who had strong social networks. Results from a mapping study including data from 17 European countries [4] showed that the lack of social support increased the risk of being exposed to physical and mental sufferings, such as cardiovascular diseases and infections.

### The *Siida*

The Norwegian state is founded on the territories of two people, the Norwegians and the Sami [7]. Today the Sami is an ethnic minority group in Norway with the status of indigenous people with their own language and cultural history [8]. In a Sami context, the term *Siida* is often used for home and community. The term means home, house, settlement, village, hamlet, moving day (certain families who move together) and home place [9]. *Siida* describes a network that consists of extended families and a working community. The term is often used in reindeer herding and describes families, relatives and other people who work together in a reindeer herding area [10]. A *Siida* network can also be settlements in a village or hamlet [11]. Based on the *Siida* tradition, many Sami families therefore include the mother, father, children, grandparents, aunts, uncles, cousins, second cousins, third cousins, godparents, godparents’ children, namesakes, and sometimes neighbours, friends and the entire community [10,12].

In cases of disease, a traditional healer is usually contacted [13]. The healer then often conducts a ritual in which he/she *reads* a prayer for healing the disease [14]. The use of traditional healers (in a Norwegian setting) is mostly known in Northern parts of Norway and is regarded as an important part of this cultural tradition. However, data from 24 communities in Norway, collected in the first survey of the SAMINOR (SAMINOR 1) study [15], found that 9.1% of the Norwegians living in these areas were familiar with and contact traditional healers in cases of disease [16]. Traditional healing is regarded as folk medicine. Folk medicine is understood as the sum total of the knowledge, skill and practices based on the theories, beliefs and experiences indigenous to different cultures, whether explicable or not, used in the maintenance of health as well as in the prevention, diagnosis, improvement or treatment of physically and mental illness [17]. In Norway traditional healing is often preserved through heritage and passed on from the older to the younger generations.

Resilience (coping, resistance and survival mechanisms) is an analytical concept which emphasises people’s health promoting processes and coping when faced with suffering and stress [18]. The term resilience was first known in developmental psychology, understood as the individual’s positive survival mechanisms [19]. Later, the concept was used in indigenous health research, in, e.g., Canada. The focus is not merely on the individual, but also on resources and processes in relationships and domestic environments in managing and coping with illness [18,20]. These scholars argue that the resilience perspective can help strengthen the contextual and cultural understanding of coping with illness. It may also help to highlight and identify socially marginalised or invisible coping strategies. Nordin-Johnsson relates resilience to the Sami traditional knowledge of which traditional healing is an important part [21]. The resilience perspective can help most people spot the resources in relationships and in the community.

Allen and colleagues studied coping strategies in indigenous youth in five circumpolar areas [22]. They found that access to support from a network of extended family and friends was an important protection factor for these youth. Nystad and colleagues [10] found that access to help and support from a network of extended family was important for developing the Sami identity and the feeling of pride. In the present study, we therefore wanted to examine the functions of the network in cases of disease.

The main research question was: What are the tasks and functions of extended networks in Northern Norway in cases of disease? We wanted to particularly examine the networks’ inner dynamics and investigate what the networks want the health personnel to know about how they handle disease. Further, we wanted to investigate the responsibility the networks take in case of disease in the family. This is so far underexplored and possibly important aspect of understanding the Sami, Kven and Norwegian inhabitants’ health practices in Northern Norway.

## Method

We employed a qualitative design based on individual and group interviews with members of networks in two communities in Northern Norway with Sami, Kven and Norwegians (mixed ethnic) inhabitants. A qualitative design is especially useful when the aim is to explore themes when limited previous knowledge exists [23]. The qualitative interview is well suited to examine the participants’ personal opinions and experiences [24]. Traditional healing is a tradition that has been preserved through silence in this culture with mixed ethnicity [13]. The first author with Sami background conducted the interviews. Necessary analytical distance (the ability to reflect upon your role as a researcher) was established by creating a research group including researchers of different professional and ethnical background [25]. We developed an interview guide based on the literature and knowledge of this culture with mixed ethnicity. This guide was used in both types of interviews. Two of the participants read and commented on the interview guide prior to collecting the data to secure *inner validity* (asked questions which provided us with the answers that is relevant to what we wanted to study) [26].

### Focus group interviews and individual semi-structured interviews

We conducted four focus group interviews: one group with five participants (*n* = 5), and three interviews with married couples (*n* = 6), in addition to 13 individual interviews. A group interview resembles natural conversations between people, and is a method that is well suited for sharing experiences about culture sensitive issues. A group of people who rely on each other may share sensitive information with the researcher, as the members of the group will protect and secure one another.

Semi-structured interviews were chosen as this design gives room for personal stories. The design also allows follow-up questions, and the participants are enabled to give nuanced answers [23]. We conducted individual interviews with 13 individuals. Some of the participants were interviewed several times. Three key informants were interviewed one to four times, due to complex dynamics. The participants were interviewed in their homes or at their workplaces. The interviews lasted 1–2 h, and were followed by drinking coffee and small talk.

### Recruitment and participants

The study took place in an area with a mixed ethnic population, included in the Sami language management area. In this area, the Sami and Norwegian languages have equal status [27]. There were approximately 2000 inhabitants in each of the two communities where this study took place [28]. The inhabitants defined themselves as Norwegian, Kven and/or Sami, and the Christian Lutheran movement Laestadianism plays a central role in the religious life of many people. According to the Sámi Parliament electoral register, 18.5% of the adult population was Sami in one of the communities and 15.5% in the other community [29]. These two communities were chosen because of already established contact through the first author’s previous research work [30].

The managers of the selected communities gave advice prior to the study and helped announce the study to the inhabitants. Information about the study was available on the communities’ websites. In addition, information about the study was published on boards in the shops, museums, office of community services and libraries. Prior to the recruitment period, an information meeting was held where interested people were provided with information. These steps to recruit participants are in line with indigenous methodology which emphasises the importance of local roots (support from the management of the communities) when conducting research [31]. The recruitment was also done through *snowball sampling*, which means that informants recommended other relevant persons to participate in the study. This method is well suited to recruit people from environments with limited access to participants.

A total of 24 participants were recruited, 12 men and 12 women. The inclusion criteria were knowledge of and experience with traditional healing; over 18 years of age; able to give an informed consent; residence in the community. The average age was 54.8 years. Sixteen participants defined themselves as Sami 1, five as Sami 2, two as mixed (Sami, Norwegian and Kven) and one as Norwegian. Nineteen participants grew up in the community and five did not. Two of the participants had senior positions in the communities, four held academic positions, three worked as office clerks, one as a farmer, two were teachers/kindergarten staff, one was a cleaner and three were canteen staff. Eight of the participants had retired. Eighteen spoke Sami and Norwegian, whereas six spoke only Norwegian. Twenty-three participants had parents or grandparents who had Sami as their domestic language. Twenty-three participants were members of the Lutheran State Church. Of these, seven had affiliation to the Laestadianism. One of the participants had no faith in religion. Twenty-two had traditional healers in the family and two had not (Table 1).

**Table 1.** Sample characteristics.

	**Total**
**Participants**	**24**
**Ethnicity**	
Sami 1 (speaking Sami or group affiliation)	16
Sami 2 (At least one parent or grandparent who speak Sami)	5
Mixed (Kven, Sami and Norwegian)	2
Norwegian	1
**Gender**	
Female	12
Male	12
**Age** Female Male	**Average: 54.8 years**50.8 years58.9 years
**Grew up in the participating community**	
Grew up in the community studied	19
Grew up outside the community studied	5
**Profession**	
Manager	2
Academician/Student	4
Office employee	3
Farmer	1
Teacher	2
Cleaner/canteen staff	4
Retired	8
**Language**	
Speak Sami and Norwegian	18
Speak Norwegian only	6
**Parents language**	
Have parents with Sami as their domestic language	21
Have grandparents with Sami as their domestic language,	2
Grandparents who did not speak Sami	1
**Religious affiliation**	
The Norwegian Lutheran State Church	16
Laestadianism and member of the Norwegian Lutheran State Church	7
Nonbeliever	1
**Traditional healer in the family**	22
**No traditional healer in the family**	2

### Ethnicity

In this study, we have chosen to classify the participants in four ethnical categories according to Bakken et al. [32]. Group 1: *Sami 1*: Those who defined themselves as Sami by speaking the Sami language, felt connected to the Sami by speaking their language, or felt affiliated with the Sami (non-ethnic group).

Group 2: *Sami 2*: Those who had at least one parent or grandparent who speak Sami, or felt a personal Sami connection (language or a feeling of affiliation).

Group 3: *Mixed ethnicity*: Those who felt affiliated with the Norwegian, Kven and/or Sami and spoke Sami, Kven or Norwegian.

Group 4: *Norwegians*: People without any Sami or Kven descendant or connection.

### Data analysis

The interviews were conducted and tape-recorded by the first author who also performed a verbatim transcription [23]. After each interview, the first author wrote down her reflections in a notebook that was later used in the analytical process. A thematic content analysis [33] was performed. The first and the last authors performed the coding. We started the process with some predefined themes from the interview guide (health personnel, healing process, what does *reading* mean to me, network in cases of serious illness, the contact, and communication between the health personnel and the healer). The themes were altered according to what emerged from the material and the research question. Three main themes including six sub-codes emerged (see the “Results” section).

The interviews were coded in NVivo 11 qualitative software [34]. Texts were assigned to different codes, which were grouped according to themes. The golden quotes (a quote that illustrates what has been abstracted) were identified from the codes, and the authors met and discussed throughout the coding process. The Sami words were first translated into Norwegian by the first author and then translated into English by a professional translator.

### Ethics, approval and consent to participate

This study meets the standard of the Helsinki Declaration of 1975 last revised in 2013 [35]. All participants were given fictitious names to sustain anonymity. Written informed consent was obtained from all participants before the interviews started, and all were informed that they could withdraw from the study for no reason. None of the participants withdrew from the study. They have all given their consent to publish.

The first author transcribed all interviews literally. After the interviews were transcribed, the participants who wished for it got the opportunity to read and approve of their own interviews (member check). Sensitive data were discussed with the participants prior to publication to secure validity [36]. Their additions were included in the interviews without changing the meaning. Some key informants were interviewed several times to ensure correct understanding of their story [23].

## Results

Based on the thematic analysis, we identified the following themes:

1. The existence and content of “traditional networks” (including the subtheme: *the importance of cultural knowledge*).
2. The role/function of these networks (including the following subthemes: *illness collective responsibility, help and comfort in difficult* times, *collective reading).*
3. What do traditional networks want health personnel to know about them and traditional healing (including these subthemes: *respect and recognition, a silent tradition, provide rooms for traditional healing).*

## Traditional networks

The participants in this study grew up with a tradition of seeking help from healers in cases of illness. Several participants also had healers in their close family or among friends. In cases of severe illness, members of the extended family (the close family, grandparents, great aunts and uncles, aunts and uncles, cousins and nephews) were often present with the patient, or they were available on the phone. An extended network of other relatives, neighbours, and friends and in some cases the entire village also provided help and support, if necessary.

### The importance of cultural knowledge

According to Martin, a participant in this study, culture competence involves showing respect for the patient´s background as well as finding out about the patient´s customs and behaviours. He said,

To respect the patient´s background, it´s all about customs and behaviors.

To exemplify, Martin tells us a story. Martin and his Sami wife had been invited to a typical Norwegian pre-Christmas party at the nursing home. This was to be a pleasant arrangement including accordion music and old songs familiar to most of the people present. His wife (who was a patient at the nursing home) was affiliated to the Laestadian congregation and not used to dance music and Christmas parties. Martin told,

This party turned into a total misunderstanding, thinking only about what the majority of the people seemed to like. But we felt miserable. Listening to deafening accordion music is against our faith. Yes, we were disgraced rather than pleased.

Short after this party, Martin and his family celebrated Christmas singing hymns and praying Our Father.

Together with some of my wife´s family, we held a short religious service, singing hymns and praying Our Father. We chose hymns that were familiar to my wife so she could hum along. That was our way of preparing for Christmas.

## The role of traditional networks

### Illness, collective responsibility

We found that the study participants thought that caring about your neighbour’s illness was a matter of common decency. Therefore, people visited the patient or they called the next of kin to find out about the patient’s condition. Martin told, “If I don’t care, it shows that I lack empathy.”

Members of the network were often present before the doctor arrived to protect and support the patient and next of kin. The neighbour’s illness affected and belonged to everyone. In many ways, it was like the network *owned* the illness. Lena explained,

What happens confirms that he’s mine. I get part of the responsibility for him. I share mine, and I share it with the rest of the society, so Stian has become a little bit ours in a way, you see. He is not only Siv’s (the mother), he becomes mine and the whole societies.

Martin claimed that Norwegians do not understand this attitude. He said,

You know he is a ”ladde” (not Sami). He does not understand anything of this, that Anna’s illness is our illness. We own the illness.

### Help and comfort in difficult times

In the period of 1860–1950, several Sami quit reindeer herding [28] and many of them settled down in the same geographical area. As residents, many Sami organised themselves in the same Siidas as they belonged to when they were nomads. Today, there are remains of this collective working system in the Laestadian congregation, which has a strong prevalence in the communities in which this study was conducted. The networks assist in conveying hope on several levels. The members visit the patients. They pray for them and comfort the families and each other. Lena tells us that her parents often prayed for the patient and also comforted and calmed the others down. The networks convey to the closest family that they are not alone. Lena tells us of a mother who was supported by the network to enable her to provide safety for her seriously ill child.

The mother feels the support of the network and that someone is backing her. She becomes a person who has her feet on the ground and thereby she dares to face her difficulties.

In cases of illness, the role of the network is to comfort and help the family with practical tasks. Members of the network do housework. They clean the floors and cook so that the next of kin can care for the patient. Some people lend their car to someone in the network who needs it. Others delegate tasks to various people in the network. Another important function is to comfort the next of kin and each other. The extended family members comfort the close family members who comfort the patient. In this way, the entire network was engaged, and the aim is to show the family that they are not alone. Martin explained, “The most important is to protect the patient and his family.” Networks that protect the family and actively take responsibility for the patient may remain from an ancient tradition. In the old days, it could take many days before the doctor arrived. Then it was important that people cared for each other. In this way, the network provides stronger solidarity between the members and affiliation to society.

### Collective reading

Another important role of the network was to contact a traditional healer (*reader*). Several participants told that in their childhood, the distance to visit a doctor was longer than to see a healer who lived in their neighbourhood. It was important for the network to be present together with the doctor to find out what diagnoses to base the *reading* on. The doctor’s diagnoses and medical assessments were important information that the members passed on to the healers. The healers used different healing prayers based on the specific medical diagnoses. In cases of less severe complaints, local and family related healers were used. Moreover, in cases of severe illness, healers from other parts of the country were contacted. If the patient was acutely ill and his life was at stake, there was interaction between the patient and the network:

There is a great interaction between the patient (the person who is severely ill) and his close relations regarding the healing process. Even though the patient doesn’t know, the network is there. (Anders)

It was usually the severity of the illness that determined the size of the network to be engaged. In the Sami and Laestadianism environments, several networks could be engaged simultaneously. Each network contacted a healer. This meant that many healers *read* for the same person. Anders explained about engaging an entire system.

P: Everyone steps in through the network. So it’s an entire system that is engaged.

In cases of severe illness, local, regional and sometimes foreign healers were contacted. Sami and non-Sami healers as well as religious and non-religious healers were used in collective *reading* for the patient. The network may also contact healers regularly through a longer period of illness, as during recovery and rehabilitation of a patient who has had a stroke. Per told that his network contacted a healer for him:

Yes, I used healers for one year, more or less intensively. Yes, I did it to regain mobility in a leg and an arm and to get my perfect vision back.

The network knows many healers. The oldest members have knowledge about the competency of the various healers. Usually some are good at *reading* for head injuries, and others for infections, and so on. One family had a notebook full of names of healers they could contact. Two participants told:

It’s about a notebook full of names … (Per)

I guess it’s this perception of what they [the healers] are good at, some are good at seeing things, and others are good at reading. (Anders)

Many of the participants in our material grew up hearing stories of healing. These healing stories were passed down from the older to the younger generation. The stories provided hope and gave younger people a recipe for dealing with illness.

The reading turns into a kind of story about coping strategies. These stories tell us that we’ll be fine and that we all have equal value and also our own resources. [It’s] a way of holding ourselves up. (Lena)

## What do networks want health personnel to know about them and traditional healing

The participants in this study thought that health personnel should have thorough knowledge about the Sami culture including traditional healing, especially those who work in communities with Sami inhabitants. Many patients want to use traditional healers for their illness and it is important that health personnel know this. Martin referred to research to explain this phenomenon:

There is this study made by Nordlandsforskning which showed that, yes, we see the doctor, but afterwards we contact a person who can read.

### Respect and recognition

In addition to knowledge about traditional healing, the participants in our study claimed that health personnel should be open and have respect for the patient’s belief and use of *reading*. Stine put it this way: “I think you should have respect for the patient or the user”. Lena emphasised the importance of balanced meetings as she explained,

Be interested in the other person. It’s about two people who have equal value, who meet and communicate.

One of the participants thought that health personnel should view traditional healing and conventional medicine as equal approaches in the treatment of illness. Traditional healing should be acknowledged as an important contribution and not be dismissed as nonsense or quackery.

I think that regardless of what you get, whether you get sugar pills or regular pills, and you get well, I think the aim must be to get well.

### A silent tradition

Many people want to talk to health personnel about *reading* because greater openness could be health promoting for the users. However, the tradition has been associated with silence. It has not been common practice to talk about the use of *reading* to people outside the closest family. Several of our participants have learnt that you should not talk openly about *reading*. Lars told us:

No, to a small extent. Sometimes I tell people when we talk about different things, but not health personnel.

Stine told us what she had learnt in this connection.

What I’ve learnt, or what mum used to say, is that you shouldn’t talk so openly about that.

The managers and health personnel in municipalities of mixed ethnicity ought to know what happened during the Norwegianisation process, and should therefore spare this group of people further sufferings. During this process, the Sami people were deprived of their identity and culture. As a consequence, many Sami people dare not tell of their culture and use of traditional medicine in fear of being perceived as superstitious and stupid. Per put it this way:

I understand that some people think it´s hard to tell in fear of being disrespected or perceived as superstitious.

The healers have also been accused of practising magic and have therefore been persecuted by the authorities for their healing practice. Nina told,

It hasn´t been common practice to tell about old Sami traditions because of the witch burning.

Several people have bad experiences from the Norwegianisation process during which the society did not recognise their culture and language. Many people were for instance physically punished or bullied when speaking Sami at school. Two participants explained,

My dad spoke Sami when he grew up, but at school he wasn´t allowed to. Those who spoke Sami were punished. (Ole)

My mom and dad didn´t know Norwegian when they started school, and they weren´t allowed to speak Sami at school. Sometimes they were also beaten for speaking Sami. (Nina)

My mom told me that they were called stupid and inferior, that they didn´t know Norwegian and weren´t able to keep up with the lessons as well as the others. (Per)

According to Norwegian law [37], many Sami and Kven people had to give up their Sami names to be allowed to purchase houses and settle down in the community. Consequently, the Sami population became invisible in society at large. Per told us,

When the Sami wanted to settle down, purchase farms and lands, they weren´t allowed unless they denied themselves and changed their family names to Norwegian names.

Today, there might be conflicts between those who know how to speak Sami and those who do not. If you do not speak Sami, you are not accepted as a real Sami, especially when you are expected to use the Sami language. Stine explained,

Then I go to meetings where most people speak Sami. That can be bad (laughing), it can be hard. I get the feeling that I should be able to, should have learned more, I should understand.

Others did not know that they were Sami before they became adults because many people denied their Sami background, as this was associated with shame and stigma.

Despite the fact that people around me spoke Sami and we have a Sami family name and the way we look, I never understood that we were Sami. (Stine)

After all, this has been associated with shame and stigma. It is important that health personnel know about the Norwegianisation process to understand the silence of the Sami population today, and to prevent further abuse and harm from occurring.

### Provide rooms for traditional networks

Furthermore, the participants wanted health personnel to facilitate for *reading*. When patients want a healer to *read* for them, this should be arranged. The participants also wanted a room where entire networks could meet. This was especially important in cases of severe illness and at the end of life. Per told us that when he was ill, he usually had many visitors.

Yes, I had lots of visitors. Sometimes my room was packed with visitors.

At the end of life, the network members want to be present day and night even though it means many people. This does not work today. One of the men explained,

The way it is today is that you have to restrict yourself from doing what you actually would have done if the person was at home.

According to the participants, greater openness about traditional healing challenges the users to put the past behind and talk openly to health personnel about the use of traditional healing. Moreover, health personnel should have knowledge about the Sami culture and the consequences following the process of Norwegianisation. Because of this process, many people have found it difficult to admit that they are Sami and that they use traditional medicine.

## Discussion

We argue that health-care professionals such as physicians, nurses, social educators, physiotherapists, paramedics and others who work with patients with Sami or mixed ethnicity will benefit from the knowledge drawn from this study. Moreover, politicians, decision-makers and health-care managers in municipalities with mixed ethnicity may consider this knowledge to improve health-care services to this group.

This study demonstrates that the patient´s network (family, kin, friends and neighbours) may play an important role when people get ill. The traditional networks in these two communities took collective responsibility for the patient as well as the relatives. The network offers help with practical tasks and supports the closest relatives. Moreover, they contact the healers. In cases of minor illness, only a few local healers are contacted. However, in cases of severe illness, local and more distant healers are contacted. This phenomenon is called *collective reading*. One of the reasons why this tradition is still practised in Northern Norway may be that many areas are still isolated, and doctors and hospitals are often far away [16].

In cases of severe illness, the participants would like the health personnel to facilitate the hospitals to accept the presence of a greater part of the network than what is presently the case. Furthermore, they called for respect for their traditions. Moreover, they wanted traditional healing to be provided to patients, upon request.

Health personnel who work in areas of mixed ethnic population should be open-minded and interested in the patients and the local culture. If they do not have knowledge about the culture, they can use colleagues, who grew up in the Sami areas, as cultural brokers. If there is no such colleague, they can ask the patients, their families or networks about what matters to the patients to provide them with integral health service. At all times, there should, for example, be a nurse of Sami background on duty. In addition, health personnel should obtain basic knowledge of the patients´ cultural background and faith. To improve the patients´ positive outcome, story-times, singing traditional hymns or duodje (traditional Sami handicraft) could be arranged for the patients in the evening. Moreover, the patients who want to attend religious services could be escorted. If the patients do not want to watch TV because of their religious faith, they should not be placed in front of the TV in the common room.

In a Scandinavian perspective, a family network is often understood as the nuclear family and the closest friends. It describes the relationship between the individual, the patient and their social network [3]. Our findings suggest that a family network in this culture with mixed ethnicity in Northern Norway is understood as an extended network, including a lot more members than what is normally the case elsewhere in Scandinavia. To describe this phenomenon, we will introduce the term “traditional network”.

The Norwegian health service is an individual-based system focusing on patients, spouses, cohabitants, brothers, sisters and parents (the closest relatives). According to Magelssen, an anthropologist and a nurse, relatives represent the patient´s home environment and social network. As informal collaborators, these people are regarded as resources in the treatment and care of the patient [38]. Previous research shows that the Sami population in Northern Norway has strong traditions of using traditional networks in cases of illness [39]. When a person is hospitalised, it is common practice to record the name of only one relative. This practice may prevent members of the traditional network from contributing actively in the healing process of the patient.

The tradition of organising large family networks is mostly known from the nomadic reindeer herders. They work together with several families in Sami homes (Siida). Such communities are found in Norway [40], Sweden [41] and Finland [42]. By the turn of the century (1800–1900), the nomadic settlements changed to other types of living. However, the Siida community was preserved and continued in the Laestadian communities which still prevails in the areas where this study was conducted [43]. See Figure 1, that illustrates the Siida home.

## Other studies

In Finland, Tervo et al. [42] found that basic traditions for healing and well-being were learned in interaction with one’s social environment. This is in line with our findings. Further, Tervo found that family and community were one of the central core values in the Sami culture. How to maintain good health and how to deal with illness were learned and guided by older people. The Siida system was based on the idea of a shared responsibility for each other within a family group. The knowledge and skills needed for this was learned and guided by the elders.

Other studies [44,45] demonstrate that the Mètis and Inuit population in Canada identified relationship with extended family members. Access to social resources and indigenous culture connections were identified as health determinants. Support from an extended family network increased their social capital. If needed, members of a network could rely on social support for oneself or other family members.

Studies among indigenous youth in Alaska show that people who are regularly in contact with an extended family network have access to more resources when they need to tackle challenges compared to people who do not have access to such a network [46]. We think that traditional networks may be a resource when you get ill. The older members in a traditional network often have thorough knowledge of who, and how many healers to contact. They also know what needs to be done in cases of illness. Historically, traditional networks have functioned as a safety net in cases of illness [12].

For more than 150 years, Norwegian authorities tried to assimilate the Sami into the Norwegian culture. Sami people felt shame, and many tried to hide their Sami ethnicity [47]. Due to the assimilation policy, many Sami traditions, the healing tradition among others, were conducted in secrecy. This policy has had, and still has, a negative effect on the health of many Sami people [48], even though studies show that the Norwegian Sami have better health compared to other indigenous people in circumpolar areas [49]. The Norwegian authorities have invested in equal access to health services and education for all people who live in Norway, including the Sami. This may explain the discrepancy in health status. In addition to equal health services and education, we believe that access to traditional networks also contributes to secure good health among the Sami in Norway.

### Resilience

Resilience may be connected to the Sami term “birgejupmi” [21]. Porsanger and Guttorm [31] understood birgejupmi as knowledge of survival capacity, and shows how people (individuals and society) in certain areas maintain their health using the resources found in the nature and in the social environment. For the participants in this study, the network was a coping resource for illness. The network provided trust and comfort. They contacted healers and prayed to God for the patient. In addition, they provided practical help like washing the floors, making food, lending out their cars and being present for the closest family so they were not left alone.

### Bourdieu and the concept of capital

The Norwegian health service is a hierarchical system in which the medical elite has monopoly on medical knowledge. The patients are treated based on a biomedical model. In this health model, health is the absence of illness. Illness is perceived as a medically understood and empirical disclosure of deviation from the health norms. This health model is associated with a model of illness that is often referred to as reductionism. This is because it is claimed that it reduces illness to complaints in consistently smaller parts of the body or to limited biological processes [50].

We can also understand traditional networks and traditional medicine as social capital in line with the French sociologist Pierre Bourdieu. According to Bourdieu, habitus (the values, faiths and norms that are consciously or unconsciously learned from the parents) can be referred to the socialisation process. In early childhood, we acquire knowledge of what is good and bad, right and wrong, possible and impossible. In this way, capital is understood as emerging from the culture in which people live and socialise. Habitus relates to how people act (gut feeling) based on their understanding of different situations [51]. Based on this, culture may be regarded as a form of habitus. According to Magelssen, culture is something that we are not generally conscious of, but something we become aware of when meeting different cultures [38]. Many of the participants in this study, for example, told that they had not reflected on their reasons for using traditional healing. It was just something they did based on their gut feeling.

Bourdieu mentions three forms of capital. Firstly, there is cultural capital that is acquired through education and competence. Secondly, there is social capital that is acquired through family relations, networks and social relationships. Thirdly, there is symbolic capital that is referred to as people´s ability to make use of the different forms of capital. Capital can be inherited. The form of capital the children get from their parents depends on what they have taught them. In this way, the establishment, organisation and use of network can be understood as social capital [51]. Many Sami children have learned how to survive and live with nature through stories. These are stories that are told in the networks and help prepare the children for adulthood. When the participants in our study learned to handle illness by using traditional medicine, this may be understood as a benefit from symbolic capital.

The capital of health personnel is the biomedical knowledge that they have obtained through their education. Patients who are ill need this knowledge, and therefore they contact the physician. To handle disease, traditional medicine is also important to many patients in Northern Norway. As long as the health personnel are not familiar with traditional medicine and networks, this use and health perspective will remain invisible and thereby represent invalid capital for them. The health personnel do not necessarily have to believe in traditional medicine or have an opinion of its effect. However, to provide these patients with integral and professional health service, it is important that the health personnel respect the patients´ wish to use health modalities that are a part of their culture [52].

The meeting between the patient and health personnel can also be regarded as a meeting between the minority and majority, in which the majority has determined what is legitimate in relation to the minority [38]. A meeting between traditional medicine and biomedical thinking may be understood as a meeting between two world views [53]. In a health professional setting, valid and invalid behaviours are defined by the health personnel by virtue of their position. As long as traditional medicine is understood as invalid capital, this use is not taken into consideration in the public health service. On the other hand, the nurse and theoretician Benner claims that health personnel is to contribute to the creation of a healing environment that supports and provide the patients with hope, as well as helps the patients make use of their social, mental and spiritual networks [54]. This may involve religion for some people. For others it may help maintain hope and strength. This perspective is in line with the guidelines for professional ethics for nurses and The Declaration of Human Rights on delivering integral professional care [52].

### Implications for practice and further research

Our studies were conducted in two coastal communities with mixed ethnicity in Northern Norway. As there are great cultural differences in this area, further research is needed to examine the importance of traditional network regarding illness in other areas with mixed ethnicity in Norway. To secure diversity, both qualitative and quantitative research methodology should be used when investigating the patients’ coping strategies in traditional networks among settlements in Norway, Sweden, Finland and Russia.

### Methodological aspects/limitations

The findings of this study should be interpreted in light of the study’s limitations. The first author is an ethnic Sami who conducts research on her own culture. One of the limitations of being familiar with the culture of the study is that you may overlook obvious elements of the culture [55]. It may also be difficult to bring forward and theorise aspects of your own culture that is regarded as sacred and secret. Our material demonstrates that there were more participants with Sami background despite the fact that both communities have a larger proportion of Norwegian citizens. The reason might be that the first author made easily contact with the Sami participants. Another reason is that the information meetings before the study start were held on the communities’ library, located in the Sami language centres that might have influenced a skewed ethnic material. Another researcher with, for instance, Norwegian or Nordic ethnic background might have received material with participants consisting of a different ethnic background. The first author discussed the aspects of her culture with her co-authors. The first author’s local roots in one of the areas studied also strengthen the study. Familiarity and knowledge of the theme have given her access to the field of research [14]. According to indigenous researcher Smith [56], you will always have a role of an outsider as a researcher, even when studying your own culture. On the other hand, the Sami cultures are not homogeneous, and may vary from place to place and develop over time. The professional background of the research group has contributed to analytical depth and distance of the data. The managers of the local communities, where the study was conducted, supported the study. This is especially important when studying indigenous people [57].

Researchers must be reflexive in all parts of the research process [58], with regard to both the theme of study and their own role in the study. According to Malterud, proximity to the research topic may complicate critical reflection [36]. However, it is our opinion that the first author´s ethnical background and previous research on the topic enable her to ask questions different from those of other researchers who did not grow up in the Sami culture. Researchers who familiarise themselves with the field have greater validity than those who do not [36]. The quality of the interviews (ask good questions) depends on the researchers´ skills and knowledge of the theme [23]. This enables them to ask more precise questions. However, if they think they know the theme and they are not curious enough to dig deeper into it, or if they are blinded by the obvious in their culture, this may naturally represent a possible source of error [59].

To become aware of her own pre-understanding and possible biases, the first author wrote a research log in which she recorded her thoughts prior to and reflections after conducting the interviews. According to Creswell, reliability can be enhanced if the researcher writes down detailed field notes [60]. In addition, all parts of the research process were discussed in the research group. Using NVivo in the analysis process enhances the transparency of all parts in the research process as well as making them more available for the other researchers in the group.

In this study, traditional networks were an extra resource that was activated by stress and illness. The network offered practical help and support, which enabled the closest relatives to be with the patient. In addition, the network contacted traditional healers to perform collective reading. Moreover, the network was essential in handling and disseminating hope and manageability on an individual as well as a collective level. Health personnel working in areas with mixed ethnicity should have thorough knowledge of this culture, including the importance of the network of these patients.
